# sigReannot: an oligo-set re-annotation pipeline based on similarities with the Ensembl transcripts and Unigene clusters

**DOI:** 10.1186/1753-6561-3-S4-S3

**Published:** 2009-07-16

**Authors:** Pierrot Casel, François Moreews, Sandrine Lagarrigue, Christophe Klopp

**Affiliations:** 1 Sigenae UR875 Biométrie et Intelligence Artificielle, Institut National de la Recherche Agronomique (INRA), BP 52627, 31326 Castanet-Tolosan Cedex, France; 2 Sigenae INRA-UMR SENAH, 35590 St-Gilles, France; 3 INRA, UMR598 génétique animale F-35000 Rennes, France; 4 AGROCAMPUS OUEST, UMR598 génétique animale F-35000 Rennes, France

## Abstract

**Background:**

Microarray is a powerful technology enabling to monitor tens of thousands of genes in a single experiment. Most microarrays are now using oligo-sets. The design of the oligo-nucleotides is time consuming and error prone. Genome wide microarray oligo-sets are designed using as large a set of transcripts as possible in order to monitor as many genes as possible. Depending on the genome sequencing state and on the assembly state the knowledge of the existing transcripts can be very different. This knowledge evolves with the different genome builds and gene builds. Once the design is done the microarrays are often used for several years. The biologists working in EADGENE expressed the need of up-to-dated annotation files for the oligo-sets they share including information about the orthologous genes of model species, the Gene Ontology, the corresponding pathways and the chromosomal location.

**Results:**

The results of SigReannot on a chicken micro-array used in the EADGENE project compared to the initial annotations show that 23% of the oligo-nucleotide gene annotations were not confirmed, 2% were modified and 1% were added. The interest of this up-to-date annotation procedure is demonstrated through the analysis of real data previously published.

**Conclusion:**

SigReannot uses the oligo-nucleotide design procedure criteria to validate the probe-gene link and the Ensembl transcripts as reference for annotation. It therefore produces a high quality annotation based on reference gene sets.

## Background

Our knowledge of genomes and transcriptomes structures is evolving quickly. The underlying idea of expression microarray is that each probe of a slide is monitoring a corresponding biological element of the transcriptome. The design is a key step of the microarray creation process. As presented by Le Brigand et al. [1] several constraints have to be taken in to account when choosing an oligo-nucleotide for a given gene. Specificity is the most important factor because it certifies the link with the element to be monitored. The second constraint is technical, the oligo-nucleotide has to be stable during the experiment and must not fold to produce a stable structure which would not be able to hybridize with the corresponding transcript. The last criterion used for species for which a large set of expressed sequence tags is available is the occurrence of the oligo-nucleotide within these tags. This eliminated oligo-nucleotides designed on transcripts which have never or seldom been monitored. The design process uses as input a set of unique sequences representing the transcriptome of the studied species. These sequences are usually cleaned of low complexity areas in order to lower the probability of cross-hybridization. The probe selection software then determines the specific sub-parts of each sequence on which the design can be performed. The software produces a number of candidate probes with the corresponding quality values. The set of input sequences has to be chosen carefully in order to represent as fully as possible the transcriptome. The quality of the selected set largely depends on the knowledge available on the studied genome. This state is closely linked to the genome assembly state and the number and variety of transcript sequences available.

New genome assemblies are produced regularly thanks to the new sequences produced in the finishing process. For each new assembly a new gene build is performed in order to locate the different transcripts on the genome and link them to a given gene. New gene builds are also produced on stable assemblies when enough new annotation is available. Finally, each gene is also under annotation by the sub-group of biologists interested in the corresponding function and can gain, lose or have its annotation modified. All these processes can impact the probe annotation: re-annotating regularly the oligo-sets is therefore highly relevant. This is the aim of sigReannot.

## Results

The results will be given on the complete oligo-set and also on a subset of 791 oligo-nucleotides which have been monitored as over or under expressed in a experiment conducted by J.M.J. Rebel from Wageningen University (unpublished).

**Figure 1:**
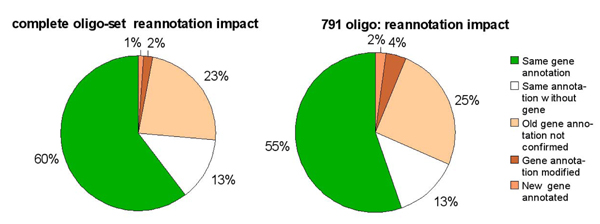
**SigReannot classes, example on the complete oligo-set**. **a) **Using the longest stretch and the global similarity criteria SigReannot classifies the oligo-nucleotides into seven categories. Oligo-nucleotides from categories 1 to 4 (in green) can be linked to a unique gene. Categories 3 and 4 have been split according to user request between oligo-nucleotides having a longest contiguous stretch larger or smaller than 30 base pairs. **b) **This figure shows the impact of the Unigene probe gene linking method on the chicken oligo-set. The gene annotation gain is 2.3% using this method.

The results presented in Figure 1 show that re-annotating with SigReannot greatly changes the gene annotation of the probes from the oligo-set. In both cases, just over half of the oligo-nucleotides are still associated with the same gene (60% and 55%) and in both cases 13% of the oligo-nucleotides could not be associated with a gene neither by the first annotation nor by SigReannot. The major difference between both annotations comes from the probes which were associated with a gene in the first annotation but which were not confirmed by SigReannot. This comes from the evolution between different versions of genome and gene builds modifying the gene sequences and transcript boundaries; nearly one quarter of the probes are in this case (23% and 25%). 2.5% of  the probes have similarity links with transcripts coming from two or more different genes and therefore cannot be uniquely assigned to a single gene by SigReannot. Some probes change or gain gene annotation (3% and 6%).

**Figure 2:**
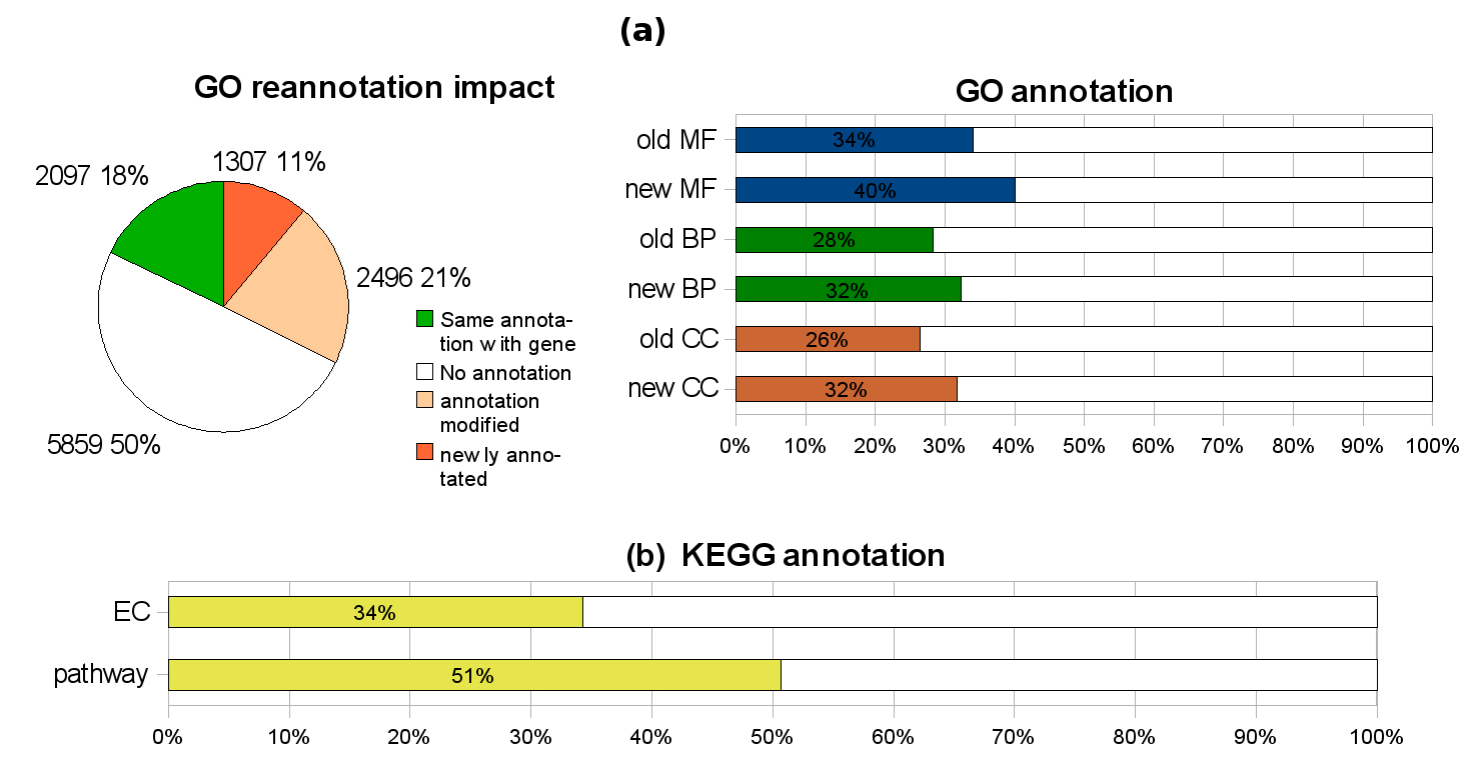
**Re-annotation impact**. The two pie charts present, for the complete set and for the previously mentioned subset, the differences between the initial annotation given by the Roslin Institute on their website and the newly generated annotation using the last chicken genome build (WUSTL 2.1) and the latest Ensembl [8] gene build of August 2006. These figures also show that the impact of re-annotation is not evenly distributed upon the probes: 31% of the probes of the sub-set versus 26% of the probes of the complete oligo-set have modified annotation.

The results presented in Figure 2a show that for a set of probes with the same gene link re-annotation permits the addition of new information. This comes from the natural enrichment of the annotation databases. GO annotation evolves following two trends, first new genes enter the ontology and second new knowledge about genes allows deeper annotation in the ontology graph. These two trends explain that one tenth of the probes have newly acquired annotation and that one fourth of the probes have modified annotation with SigReannot.

In Figure 2b, the KEGG annotation version of the EADGENE chicken oligo-nucleotide set was used to re-analyse data previously published (desert et al [2]) and corresponding to the gene clusters down- or up- regulated after 16 h fasting compared to the fed states in chicken liver. Three additional Kegg pathways with a minimum of 3 genes associated (see desert et al [2] for their selection) were found for these two gene clusters. It concerns "Glycolysis.Gluconeogenesis", 'Galactose.metabolism', 'Pyruvate.metabolism' for the down- regulated gene clusters and, "Pentose.phosphate.pathway", "Fructose.and.mannose.metabolism", "Alanine.and.aspartate.metabolism" for the down- and up- regulated gene clusters respectively.

## Methods

The pipeline chains three steps. The first step tries to link each oligo-nucleotide to a given gene, the second step retrieves from different sources functional annotation using the gene identifier and the last step formats the data in several files corresponding to the biologists' needs.

### Step 1: Linking each oligo-nucleotide to a gene

The aim is to verify, if the design criteria are still matched. The specificity of the oligo-nucleotide is verified by aligning it versus a set of existing transcripts. Transcript files are produced by the Ensembl [3] and the NCBI teams for most sequenced genomes. SigReannot uses two Ensembl transcript files  containing all known cDNAs and non-coding RNAs.

The association is simply based on similarity criteria which can be calculated using a blast [4] output. These criteria have been determined experimentally through correlating similarity values with hybridization results. SigReannot uses criteria given by Liebich and Schadt et al. [5] Kane et al. [6] and He and Wu et al. [7]. Two alignment criteria are taken into account to link an oligo-nucleotide to a transcript. The first one is the longest contiguous stretch. As soon as this one is longer than 15 base pairs for 50 mers (20 bp for 70 mers), a low quality link, or noise link, will be registered between the oligo-nucleotide and the transcript. And the second one is the global identity percentage, if this criteria is higher than 85%, then a high quality link, or good hit, will be registered between the oligo-nucleotide and the transcript. This criterion is computed dividing the number of nucleotides matching between the transcripts and the oligo-nucleotide by the length of the oligo-nucleotide.

**Figure 3:**
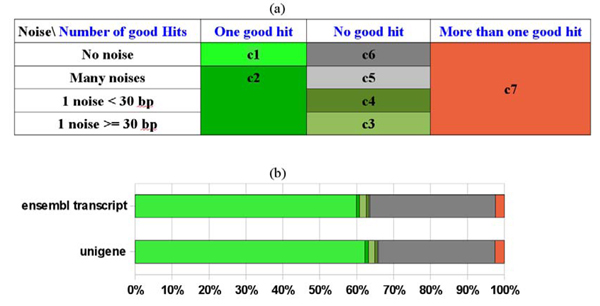
**Re-annotation impact on GO annotation and KEGG annotation**. **a) **This diagram is build on the 12,351 probes having the same gene annotation initially and with SigReannot. In both cases the Gene Ontology annotation was extracted and compared. **b) **The initial annotation file doesn't give Kegg annotation thus, no comparison can be done.

Using these two types of links SigReannot divides the oligo-nucleotides in 7 classes defined in the Figure 3a.

To find more oligo-gene link, the pipeline also uses the results of sequence similarity searches versus the Unigene clusters [8]. Ensembl uses stringent thresholds in the gene build process and produces often short UTRs. Manual probe re-annotation has shown that in some cases the design of the probe was done in an area very close but outside of the Ensembl selected UTR region of the transcript or in an intron. This comes first from the sequences selected to design the probes which are often ESTs presenting splice differences, and second from the high weight of the specificity criteria in the probe selection process. In order to maximize the number of probes with annotation, the pipeline checks if a probe has a similarity with a unique Unigene cluster and if this cluster can be uniquely linked to an Ensembl gene. In this case the pipeline extracts an extended region (1000 bp up and downstream) around the transcript to locate the probe. If these steps succeed then the oligo-nucleotide is linked to the corresponding gene and its category is updated. All oligo-nucleotides from categories 3 to 6 will undergo this processing step (see Figure 1b for the impact). Once each oligo-nucleotide is classified, probes from classes 1 to 4 will be functionally annotated.

### Step 2: retrieving annotation using the Ensembl API (Application Programming Interface)

Once an oligo-nucleotide is linked to a gene, the Ensembl API enables the corresponding human, mouse and rat orthologuous genes (Ensembl gene ID, HGNC and its description), the GO identifiers for each Gene Ontology category (ID, class, evidence code and definition) and the external references (database_name and the xref ID) to be fetched. Then, using the human HGNC ID of the Ensembl gene other annotations are fetched from the KEGG database:

• Pathway ID and description.

• KEGG genes: KO ID, EC, gene ID and definition for the annotated species, human, mouse and rat.

These informations are stored in a local Mysql database.

### Step 3: data formatting

Once all the annotation is stored in the database, the aim is to extract them into a user-friendly format for biologists. According to their demands, these data are extracted to comma separated files commonly opened within a spreadsheet. Using these data SigReannot also generates correspondence matrices linking each oligo-nucleotide, in rows, to its GO term, in columns. The junction of a row and a column equals one if the oligo-nucleotide has this annotation, and zero if not.

In the current version of SigReannot eight files are provided.

For the EADGENE oligo-sets, these files can be downloaded from the network website at [9].

The oligo-set used in this paper was designed in 2005 by the Roslin Institute and contains 20 460 oligo-nucleotides. The chicken oligo-nucleotides were designed against a mixed panel of ESTs from Genbank/EMBL, Ensembl release 30 genes and transcripts, UMIST chicken ChEST cDNAs, miRBase RNAs and contributed sequences. The initial annotation file can be downloaded from: .

## Discussion

One element which has been thoroughly discussed with the users and other teams working on tools with the same aim is the impact of the alignment strand on the annotation. Some probes of the EADGENE oligo-sets have obviously been designed on the opposite strand of the gene. With the usual transcript extraction protocol these probes should show no signal because the transcripts should not hybridize. However some of these probes are measured as under or over expressed in experiments. This may be the result of hybridization with a part of the genome which is not sequenced yet or else the result of antisense transcription of these genes. More and more evidence [10] supports the conclusion that quite a lot of transcripts have also antisense expression. Therefore SigReannot annotates probes with the corresponding gene and the strand. Analysing manually the localization of the probes on the genome showed that some of them were designed in intronic regions. This comes from the fact that the splicing machinery does not always perform in the same way. For these probes it is possible to calculate another quality criteria which would be the ratio of the unspliced EST over the spliced EST at this location. This criteria would express the probability of monitoring the expression of the gene using this probe. With a large number of ESTs from different conditions it would be possible to specify the criteria following that condition. To finish, the binding free energy criteria often mentionned in the oligonucleotide design paper is not used in this version of SigReannot.

## Conclusion

Because microarray oligo-sets design is expensive and time consuming, and because the biologists community is willing to share its results, oligo-sets are often used for several years. During this time, the genome assembly quality and the amount of annotation are increasing. These elements explain why biologists are interested in up-dated annotation for existing oligo-sets. The main novelty of sigReannot is to provide biologists with quality criteria about the annotation, letting them decide how to exploit it. Even if the microarray technique is questioned with the arrival of the new sequencing technologies, pipelines like SigReannot will be relevant infrastructures to link long SAGE [10] tags with the corresponding transcripts.

## Competing interests

The authors declare that they have no competing interests.

## Authors' contributions

SL specified largely and tested the pipeline. PC and CK developed SigReannot. PC analysed the data. FM contributed to the KEGG annotation part of SigReannot. PC and CK drafted the manuscript. All authors read and approved the final manuscript.
